# Impact of yellow fever virus envelope protein on wild-type and vaccine epitopes and tissue tropism

**DOI:** 10.1038/s41541-022-00460-6

**Published:** 2022-03-23

**Authors:** Emily H. Davis, Binbin Wang, Mellodee White, Yan-Jang S. Huang, Vanessa V. Sarathy, Tian Wang, Nigel Bourne, Stephen Higgs, Alan D. T. Barrett

**Affiliations:** 1 Department of Pathology, University of Texas Medical Branch (UTMB), Galveston, TX USA; 2 Sealy Institute for Vaccine Sciences, UTMB, Galveston, TX USA; 3 Department of Pediatrics, UTMB, Galveston, TX USA; 4 Biosecurity Research Institute, Kansas State University, Manhattan, KS USA; 5 Department of Diagnostic Medicine/Pathobiology, College of Veterinary Medicine, Kansas State University, Manhattan, KS USA; 6 Center on Emerging and Zoonotic Infectious Diseases, College of Veterinary Medicine, Kansas State University, Manhattan, KS USA

**Keywords:** Preclinical research

## Abstract

The envelope (E) protein of flaviviruses is functionally associated with viral tissue tropism and pathogenicity. For yellow fever virus (YFV), viscerotropic disease primarily involving the liver is pathognomonic for wild-type (WT) infection. In contrast, the live-attenuated vaccine (LAV) strain 17D does not cause viscerotropic disease and reversion to virulence is associated with neurotropic disease. The relationship between structure-function of the E protein for WT strain Asibi and its LAV derivative 17D strain is poorly understood; however, changes to WT and vaccine epitopes have been associated with changes in virulence. Here, a panel of Asibi and 17D infectious clone mutants were generated with single-site mutations at the one membrane residue and each of the eight E protein amino acid substitutions that distinguish the two strains. The mutants were characterized with respect to WT-specific and vaccine-specific monoclonal antibodies (mAbs) binding to virus plus binding of virus to brain, liver, and lung membrane receptor preparations (MRPs) generated from AG129 mice. This approach shows that amino acids in the YFV E protein domains (ED) I and II contain the WT E protein epitope, which overlap with those that mediate YFV binding to mouse liver. Furthermore, amino acids in EDIII associated with the vaccine epitope overlap with those that facilitate YFV binding mouse brain MRPs. Taken together, these data suggest that the YFV E protein is a key determinant in the phenotype of WT and 17D vaccine strains of YFV.

## Introduction

Yellow fever virus (YFV) is the prototype member of the genus *Flavivirus* and of great importance to global public health. The virus is endemic to sub-Saharan Africa and tropical South America where each year an estimated 170,000 cases of severe yellow fever (YF) lead to approximately 60,000 deaths^1^. YF is a viscerotropic disease characterized by hemorrhagic fever and multiorgan failure resulting from extensive damage to the liver, kidneys, and heart. Supportive care is the only option for those that present with YF since there are no approved antivirals for the treatment of any flavivirus disease. Prophylactically, YF is controlled by a live-attenuated vaccine (LAV), termed 17D.

The YFV 17D vaccine strain was derived from the wild-type (WT) strain Asibi, originally isolated from a mild case of human YF (it is named after the Ghanaian man from which it was isolated). The 17D strain was empirically derived by 176 serial passages in mouse and chicken tissues^2^. During serial passage in chick embryos lacking neuronal tissue, the virus lost its ability to cause viscerotropic disease in monkeys and could no longer be transmitted by mosquitoes. The resultant attenuated strain has been used successfully for over 80 years and is considered to be one of the most effective viral vaccines. Today the 17D vaccine is actually used as three substrains (17D-204, 17D-213, and 17DD) all derived from the original 17D vaccine, which is no longer available^3^. Phenotypically, all three vaccine substrains are indistinguishable in vaccinees and are regarded as equally safe and efficacious.

Concurrent with the development of the 17D virus, another YF LAV, the French neurotropic virus (FNV), was also generated^4,5^. The FNV virus was derived through serial passage in mouse brain, which resulted in it losing the ability to cause viscerotropic disease in monkeys.

In terms of pathogenicity, WT YFV causes viscerotropic disease in primates with the liver being the primary site of disease. Interestingly, even if the virus is administered in the brain of non-human primates, the animals succumb to viscerotropic rather than neurotropic disease^6^. In contrast, WT YFV causes neurotropic disease in immunocompetent mice. The 17D substrains also cause neurotropic disease in immunocompetent mice, and very rarely in primates (including humans); however, they do not cause viscerotropic disease in any host. However, infection by WT YFV and the 17D vaccine leads to mortality in immunocompromised interferon-αβγ receptor knockout (AG129) mice by different mechanisms, as WT YFV infects the liver but not the brain, while 17D virus is vice versa^7^.

**Fig. 1:**
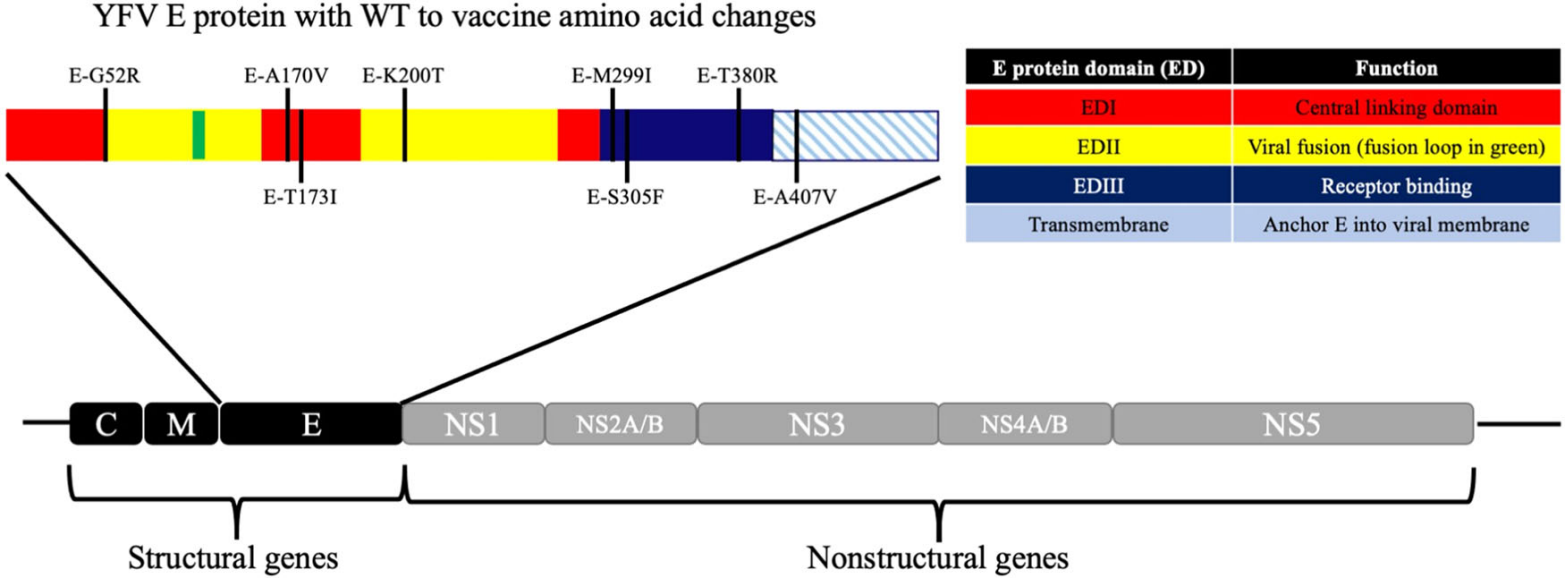
Structure of the YFV genome and E protein. The YFV genome is co-translationally cleaved by host and viral proteases. The E protein is displayed with amino acids that differentiate the WT YFV strain Asibi from the YFV vaccine strain 17D marked.

The flavivirus genome encodes 10 genes that are translated as a single polyprotein that is co-and post-translationally processed into three structural proteins that make up the viral virion (capsid (C), membrane (M), and envelope (E)) and seven nonstructural (NS) proteins (NS1, NS2A, NS2B, NS3, NS4A, NS4B, and NS5) that form the replication complex. The flavivirus lifecycle begins with the attachment of the virus to the host cell, a process that is mediated by the interaction of the viral E protein with as yet to be identified host receptor(s). The E protein N-terminal ectodomain contains three domains (EDI, EDII, and EDIII) and a transmembrane stem-anchor region. Of the 20 amino acids that distinguish WT Asibi from the 17D vaccine, eight reside in the E protein and one in the membrane protein, underscoring the importance of the structural proteins to attenuation (Fig. 1).

Previous studies have investigated the structure-function relationship of the YFV E protein using monoclonal antibodies (mAbs)^8–17^. These studies identified E protein epitopes that are either WT YF-specific (mAbs 117, S17, S18, S24, and S56), vaccine-specific (17D-204, 17DD, 17D-213, and FNV; mAbs 411 and H5), 17D-204 vaccine substrain-specific (mAbs 864 and 8A3), 17DD vaccine substrain-specific (mAb H6), or FNV-specific (mAb 429). Most of these mAbs have low or no neutralizing activity and so to date there has been relatively little characterization of their target epitopes. One exception is the 17D-204 vaccine substrain-specific epitope identified using mAb 864, which elicits high neutralizing activity allowing for mAb neutralization resistant mutants to be generated. Using these techniques mAb resistance was mapped to the epitope that include residues E-305 and E-325 on EDIII^18^.

In this paper, we map WT-specific mAb 117 and vaccine-specific mAb 411 on the surface of the YFV E protein with respect to WT and vaccine-specific epitopes using a combination of fluorescent microscopy and chimeric 17D and Asibi infectious clone (i.c.)-derived viruses with substitutions in the E protein. In addition, we have previously adapted a technique developed to investigate the binding of ligands to neurotransmitters in mouse and rat brain neuronal plasma membrane receptor preparations (MRPs)^19^ as an in vitro system for identifying interactions of YF strain FNV, Japanese encephalitis, West Nile and tick-borne encephalitis viruses to MRPs derived from mouse and monkey brain^20–24^. In the studies reported here, we have optimized the MRP technique using liver and brain from AG129 mice and incorporated mouse lung as a non-virus-binding organ control to ensure biologically relevant results. Using mAbs, fluorescent microscopy, MRPs, and chimeric i.c-derived viruses, we show that the residues involved in WT and vaccine-specific E protein epitopes defined in this study overlap with the residues that give YFV the ability to bind mouse liver and brain, respectively. Based on these data, it is suggested that the YFV E protein is a key determinant in the phenotype of WT and LAV strains of YFV.

## Results

### Generating YFV i.c.-derived viruses with M or E protein mutations

To investigate the effect of E protein mutations on WT and vaccine epitopes, chimeric Asibi and 17D i.c. derived viruses were generated by exchanging prM/E genes, EDIII, single-site amino acid residues at every M and E protein residue that differentiates Asibi and 17D plus 17D-204 specific residue E-325^25–27^. The M protein was included in the panel of mutants as it has been shown to influence the flavivirus lifecycle, including maturation of the E protein^28,29^. Thus, single-site mutants were generated at residues M-36, E-56, E-170, E-173, E-200, E-299, E-305, E-325, E-380, and E-407 of both Asibi and 17D i.c. viruses (Table 1). All recombinant viruses were recovered with the expected mutations following transfection of Vero cells, and no compensatory mutations were detected in the consensus sequences indicating that any changes in binding are due to the residues mutated.

**Table 1:** Single-site changes to structural proteins affect the way WT and vaccine-specific mAbs bind YFV.

			MAB 117		MAB 411		LIVER MRP				BRAIN MRP			
	Amino acids		Virus backbone		Virus backbone		Asibi backbone		17D backbone		Asibi backbone		17D backbone	
RESIDUES	Asibi	17D	Asibi	17D	Asibi	17D	Log_10_ change	Significance (*p*-value)	Log_10_ change	Significance (*p*-value)	Log_10_ change	Significance (*p*-value)	Log_10_ change	Significance (*p*-value)
*FULL LENGTH*			+	−	−	+	1.8	<0.0001	0.4	0.25	0.4	0.98	1.9	0.0023
*PRME*			−	+	+	−	0.1	0.99	2.1	<0.0001	1.4	<0.0001	0.3	0.21
*EDIII*			+	−	+	−	0.4	0.23	0.04	0.98	1.1	<0.0001	0.08	0.87
*M-36*	L	F	−	+	−	−								
*E-52*	G	R	+	+	−	+	0.2	0.35	1.1	<0.0001	0.4	0.013	1.1	<0.0001
*E-170*	A	V	−	+	−	+	0.09	>0.9999	1.4	<0.0001	0.8	<0.0001	0.4	0.04
*E-173*	T	I	−	+	−	+	0.2	0.34	0.9	<0.0001	0.09	0.81	0.2	0.35
*E-200*	K	T	+	+	−	+	1.6	<0.0001	0.5	0.0021	1.6	<0.0001	0.5	0.0021
*E-299*	M	I	+	+	+/−	−	0.6	0.0003	1.1	<0.0001	1.5	<0.0001	0.7	<0.0001
*E-305*	S	F	+	−	+	−	1.9	<0.0001	0.1	0.62	3.3	<0.0001	0.4	0.047
*E-325*	P	S	+	−	+	−	0.7	0.0001	0.2	0.53	1.1	<0.0001	2.2	<0.0001
*E-380*	T	R	+	−	+	−	1.4	<0.0001	0.2	0.25	2	<0.0001	3.3	<0.0001
*E-407*	A	V	−	+/−	+	+	1.5	<0.0001	2.5	<0.0001	0.5	0.0083	0.2	0.44

Amino acid changes that differentiate Asibi and 17D are listed. The ability of WT (mAb 117) and vaccine (mAb 411) specific mAb was tested using structural chimeras of both Asibi and 17D viruses and immunofluorescent microscopy. Positive (+), negative (−), and equivocal (+/−) fluorescence.

### Mapping YFV WT and vaccine-specific epitopes on the structure of the E protein

Because the WT and vaccine epitopes recognized by mAbs 117 and 411, respectively, have received limited characterization to date and both were identified by screening of virus-infected cells by indirect immunofluorescent staining we decided to use fluorescence microscopy, rather than ELISA, to investigate mAb binding^9,30,31^. WT and vaccine-specific epitopes were mapped onto the structure of the E protein using immunofluorescent microscopy of Vero cells infected with chimeric Asibi and 17D i.c.-derived viruses described above. Asibi and 17D i.c. viruses were used as controls.

**Fig. 2:**
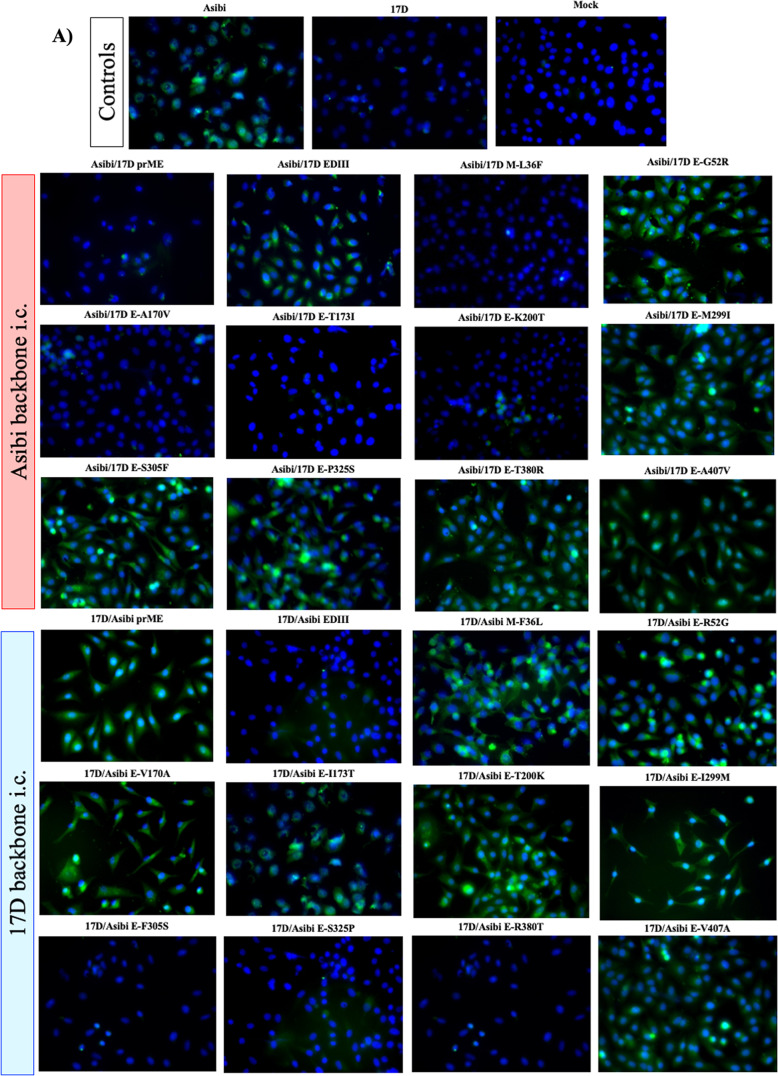

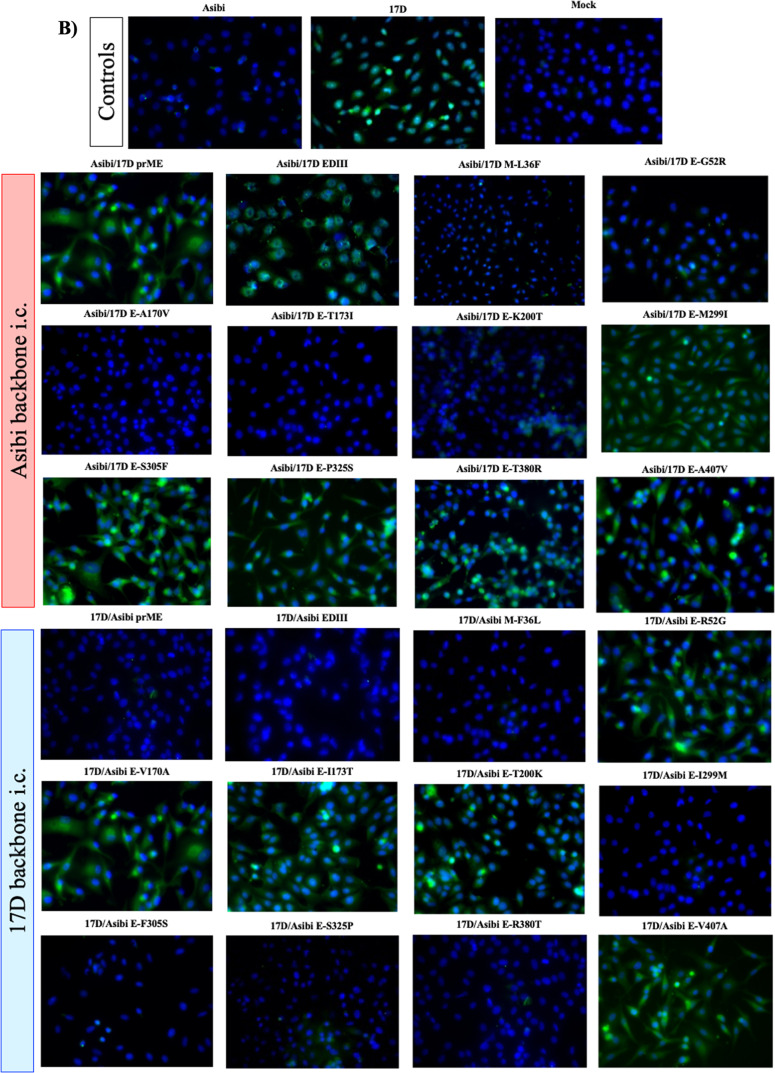
Structural genes of YFV are responsible for binding of YFV to WT-specific mAb 117 and vaccine-specific mAb 411. The WT epitope of mAb 117 was mapped using YFV structural chimeras of Asibi and 17D viruses (**A**). The WT epitope of mAb 411 was mapped using YFV structural chimeras of Asibi and 17D viruses (**B**).

WT-specific mAb 117 displayed a filamentous binding pattern, with distinct punctae (Fig. 2a) as reported previously^11^. MAb 117 did not bind to cells infected with Asibi virus containing 17D prM/E genes, but bound when either 17D EDIII or M-36 alone were exchanged (Fig. 2a, Table 1). This suggests that EDI and/or EDII plus M contain residues either directly or indirectly involved in the epitope recognized by mAb 117 (Fig. 2a, Table 1). In comparison, MAb 411 binding to 17D virus-infected cells was characterized by a diffuse staining outside of the nucleus (Fig. 2b, Table 1), also as reported previously^11^. MAb 411 lost specificity to 17D when either Asibi prM/E or EDIII swaps were made (Fig. 2b, Table 1); but not when M-36 alone was changed (Fig. 2b, Table 1). This suggests that the epitope recognized by vaccine-specific mAb 411 is located in E and has a strong association with EDIII (Fig. 2b, Table 1).

The panel of chimeric Asibi i.c. and 17D i.c. single-site mutants was used to further define the residues involved in the WT and vaccine-specific epitopes. When individual amino acids were exchanged and tested against WT mAb 117, changes at E-170, E-173, or E-200 to 17D in the Asibi backbone abolished mAb binding (Fig. 2a, Table 1). In the 17D backbone, reversal of individual residues E-52, E-170, E-173, E-200, E-299, and E-407 to Asibi residues allowed mAb 117 to bind the vaccine strain (Fig. 2a, Table 1). Together these results strongly suggest that EDI and EDII contain the critical residues of the WT-specific epitope recognized by mAb 117.

Similarly, when the single residue mutated chimeras were used to define the vaccine-specific epitope recognized by mAb 411, reversion of residues E-299, E-305, E-325, and E-380 to Asibi in a 17D i.c. backbone abolished binding whereas exchange of residues E-299 and E-305, E-325, and E-380 to 17D in the Asibi backbone allowed the vaccine-specific mAb to bind (Fig. 2b, Table 1) indicating that the critical residues in the vaccine epitope were located in EDIII.

### Mapping of WT and vaccine epitopes onto the structure of the E protein

**Fig. 3:**
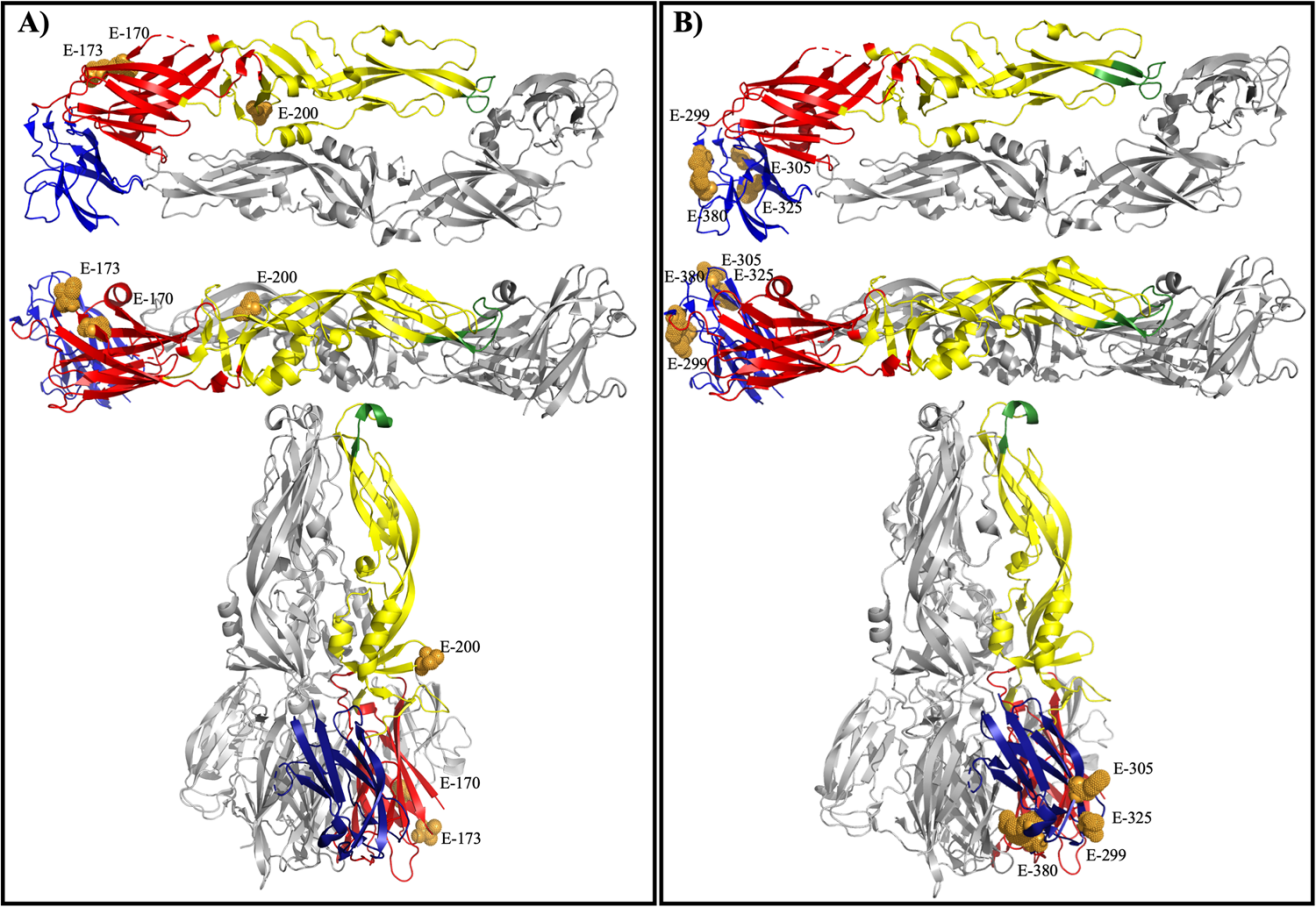
WT-specific epitope mapped to YFV EDI/EDII of the E protein and vaccine-specific epitope mapped to YFV EDIII. Residues important to the mAb 117 epitope in both Asibi and 17D backbones were mapped to the prefusion E protein structure and displayed from the top and side view and post-fusion structure (**A**). Residues important to the mAb 411 epitope in both Asibi and 17D backbones were mapped to the prefusion E protein structure and displayed from the top and side view as well as the post-fusion structure (**B**).

When the residues (E-170, E-173, and E-200) involved in binding of WT-specific mAb 117 were mapped onto the prefusion YF E protein, it is clear that the epitope is exposed on top of the mature virion (Fig. 3A). Specifically, E-173 extends beyond the virion surface, which is clearly visible on prefusion E protein (Fig. 3A). E-170 is located near E-173 but does not extend past the surface of the protein. E-200 is located in the middle of the dimerization domain, near the surface of the virion. In the post-fusion structure, the residues responsible for a change in binding are exposed on the outside of the trimer spike (Fig. 3A). E-173 and E-200 are readily accessible, whereas E-170 is located further from the surface of the trimer.

When the residues involved in binding of 17D vaccine-specific mAb 411 were mapped onto the prefusion YF E protein, it also showed that the epitope is exposed on the outside of the virion (Fig. 3B). E-299, E-305, E-325, and E-380 are located on the upper lateral ridge of EDIII with E-305 being the most readily accessible (Fig. 3B). In the post-fusion structure, E-299 and E-380 are buried behind E-305 and E-325, which are both exposed at the bottom of the trimer spike (Fig. 3B).

### Binding of WT Asibi and 17D vaccine virus to MRPs derived from AG129 mice

In AG129 mice WT YFV causes viscerotropic disease, i.e., infection of the liver and not the brain, while 17D vaccine infects the brain and not the liver^7^. Therefore, we investigated the binding of WT Asibi and 17D vaccine viruses to MRPs derived from AG129 mouse tissues. To facilitate interpretation of the results, binding data are presented as log_10_ reduction in infectivity in MRP samples when compared to controls, fold change in infectivity titer, and percent loss in infectivity titer (i.e., percentage of infectious virus bound to MRPs).

Initial studies investigated the specificity of binding of biological (i.e., non-infectious clone) Asibi and 17D-204 viruses to MRPs derived from liver and brain tissues, and to MRPs derived from lung, a non-YFV tropic organ. Thus, we could determine whether or not binding of virus to MRPs was due to non-specific interactions (Supplementary Table 1). Asibi virus bound to AG129 liver MRPs (1.4 log_10_ reduction [equivalent to 26.5-fold, or 96.2% of virus infectivity], *p* < 0.0001) but not to brain (0.2 log_10_ reduction [equivalent to 1.6-fold, or 35.5% of virus infectivity], *p* = 0.99) nor lung MRP (0.05 log_10_ reduction in titer or 0.9-fold, or 11.9% of virus infectivity, *p* = 0.99). In comparison, 17D-204 did not bind either liver (0.7 log_10_ change (equivalent to 4.6-fold, or 78.1% of virus infectivity, *p* = 0.22) nor lung MRP (0.2 log_10_ reduction in titer or 0.7-fold, or 33.0% of virus infectivity, *p* = 0.22). but titer was reduced 2.2 log_10_ (equivalent to 158-fold, or 99.4% of virus infectivity, *p* < 0.0001) with brain MRP. In summary, neither virus bound lung MRP, WT Asibi bound liver but not brain, and 17D-204 vaccine vice versa. These results indicated that the MRP technique could be used to investigate relative binding of the different viruses used in these studies. It was decided that for the rest of the MRP studies results would be shown as log_10_ reduction only; however, data for fold change and percent reduction of viral titer are shown in Supplementary Table 1. In addition, it was decided that ≤1.0 log_10_ reduction in infectivity titer, equivalent to 10-fold, or 90% of virus infectivity binding to the MRP, would not be considered biologically significant as Asibi bound liver and 17D bound brain at ≥1.0 log_10_ reduction.

Asibi and 17D i.c.s were tested in the AG129 MRP system to establish whether or not these recombinant-derived viruses behaved like their biological counterparts (Supplementary Table 1). Like the biological Asibi virus, Asibi i.c. was able to bind liver MRP (1.8 log_10_ reduction, *p* < 0.0001) but did not bind either brain MRP (0.4 log_10_ reduction, *p* = 0.98) nor lung MRP (0.6 log_10_ reduction, *p* = 0.46). 17D i.c. also resembled its virus counterpart as it bound to brain MRP (1.9 log_10_ reduction, *p* < 0.0023) but not liver MRP (0.4 log_10_ reduction, *p* = 0.22) nor lung MRP (0.6 log_10_ reduction, *p* = 0.25). Notably, binding of Asibi and Asibi i.c. to liver and 17D and 17D i.c. to brain each differed <0.3 log_10_, or 2-fold, showing binding of biological and recombinant viruses was indistinguishable.

### Plaque picks of MRP-resistant Asibi and 17D viruses displays changes to the E protein

To confirm the binding data, unbound virus remaining in the supernatant of MRP assays was examined. The free virus was plaque-picked (p.p.) in order to assess its amino acid identity. RNA from these MRP- resistant (MRP^R^) viruses was isolated and structural genes sequenced (Supplementary Table 2). Two p.p. were isolated from liver MRP supernatant of both Asibi and 17D i.c.s and three p.p. were isolated from brain MRP supernatant. In all three Asibi liver MRP^R^ viruses, residues changes were detected at E-A56V, E-A170V, E-T173I, and E-K200T, all representing a change to 17D residues including E-56, which is present in two out of three 17D substrains, namely 17D-204 and 17D-213. 17D liver MRP^R^ viruses reported a conserved E-V56A change, which represents a reversion to Asibi. One E protein amino acid change was recorded in one of three Asibi brain MRP^R^ viruses at E-S349T.

### Ratio of viral RNA per pfu confirm that reduction in infectivity titer is due to virus binding MRPs, not a difference in numbers of viral particles

Next, we determined if the MRP binding data were influenced by a difference in the particle to infectivity (pfu) ratios of Asibi and 17D. In order to test the contribution of viral infectivity to titer, qRT-PCR of cell culture supernatant of viruses used in the MRP assays was undertaken to define viral RNA copies per pfu for biological and i.c. derived Asibi and 17D viruses. Using capsid gene primers of a region that is identical between Asibi and 17D, the vRNA per pfu ratio were statistically indistinguishable (Asibi: 37 copies of vRNA per pfu (vRNA/pfu), Asibi i.c.: 26 vRNA/pfu, 17D-204: 24 vRNA/pfu, and 17D i.c.: 30 vRNA/pfu) suggesting the change in titer was indeed due to MRP binding.

### YFV interaction with MRP is determined by E protein

In order to determine if the E protein drives the interaction of YFV with mouse organ MRPs, Asibi and 17D prM/E chimeras were examined for their ability to bind to AG129 mouse brain and liver MRPs (Table 1). Asibi i.c. with 17D prM/E did not bind to liver MRPs (0.1 log_10_ reduction, *p* = 0.99), whereas Asibi i.c. did (1.8 log_10_ reduction, *p* < 0.0001). As expected, Asibi i.c. with 17D prM/E genes bound brain MRPs (1.4 log_10_ reduction, *p* < 0.0001) whereas Asibi i.c. did not (0.4 log_10_ reduction, *p* = 0.98). 17D i.c. with Asibi prM/E did bind to liver MRPs (2.1 log_10_ reduction, *p* < 0.0001) whereas 17D i.c. did not (0.4 log_10_ reduction, *p* = 0.25). Similarly, 17D i.c. with Asibi prM/E genes did not bind brain MRPs (0.3 log_10_ reduction, *p* = 0.21) whereas 17D i.c. did (1.9 log_10_ reduction, *p* < 0.0001). These data demonstrate that, at least in AG129 mice, the structural genes are responsible for tissue binding specificity of WT Asibi and 17D-204 vaccine viruses.

EDIII is regarded as the putative receptor-binding domain of flaviviruses^30–37^, although no receptor-binding molecule has been conclusively identified. In order to determine if this domain was involved in the interaction of Asibi and/or 17D viruses with AG129 MRPs, EDIII was swapped in the i.c.-derived viruses, and the ability of these chimeric viruses to bind liver and brain MRPs was tested (Table 1). Asibi i.c. with 17D EDIII did not bind liver MRP (0.4 log_10_ reduction, *p* = 0.23) but bound brain MRP (1.1 log_10_ reduction, *p* < 0.0001). 17D i.c. with Asibi EDIII was unable to bind liver MRP (0.04 log_10_ reduction, *p* = 0.98) or brain MRP (0.08 log_10_ reduction, *p* = 0.87). When compared to the control data for Asibi i.c. and 17D i.c. presented above (Supplementary Table 1), these data show that EDIII is responsible for how 17D i.c. binds to AG129 mouse brain MRPs.

### Individual residues affect how YFV binds mouse liver MRPs

To obtain a more detailed understanding of E protein residues involved in binding to AG129 mouse liver MRPs we utilized our panel of Asibi and 17D i.c.-derived chimeric viruses. Exchanging the 17D residues E-52, E-170, E-173, and E-299 for those in the Asibi backbone, resulted in a loss of binding [E-52 (0.2 log_10_ reduction, *p* = 0.35), E-170 (0.09 log_10_ reduction, *p* > 0.99), E-173 [0.2 log_10_ reduction, *p* = 0.34), and E-299 [0.6 log_10_ reduction, *p* = 0.003) and induced binding to mouse liver MRPs when exchanged in the 17D backbone [E-52 (1.1 log_10_ reduction, *p* < 0.0001), E-170 (1.4 log_10_ reduction, *p* < 0.0001), and E-299 [1.1 log_10_ reduction *p* < 0.0001) (Table 1). As residue E-170 had the largest effect in both backbones, it appears critical to binding of Asibi virus to mouse liver MRPs and the E-52 and E-299 contribute to binding peripherally. The binding data for E-173 in 17D i.c. backbone (0.9 log_10_. *p* < 0.0001) was considered equivocal based on our 1.0 log_10_ cut-off, The importance of all three domains to binding of AG129 liver MRPs suggesting the binding site is conformational rather than a linear sequence.

The exchange of 17D E-325 did not significantly ablate the ability of Asibi virus to bind AG129 liver (0.7 log_10_ reduction, *p* = 0.001) by our definition of binding nor did it affect ability of 17D to bind liver (0.2 log_10_ reduction, *p* = 0.53). The data for Asibi/17D E-325 was equivocal. Though statistically significant, the virus bound <1.0 log_10_.

The exchange of 17D E-407 did not affect the ability of Asibi to bind liver (1.5 log_10_ reduction, *p* < 0.0001), but introduction of the Asibi residue did result in binding of 17D to mouse liver MRP (2.5 log_10_ reduction, *p* < 0.0001).

17D residues E-200, E-305, and E-380 did not impact Asibi binding to mouse liver MRP [E-200 (1.6 log_10_ reduction, *p* < 0.0001), E-305 (1.9 log_10_ reduction, *p* < 0.0001), and E-380 (1.4 log_10_ reduction, *p* < 0.0001)] nor enable 17D to bind mouse liver MRP [E-200 (0.5 log_10_ reduction, *p* = 0.0021), E-305 (0.1 log_10_ reduction, *p* = 0.62), and E-380 (0.2 log_10_ reduction, *p* = 0.26)].

### Individual residues affect how YFV binds mouse brain MRPs

To obtain a more detailed understanding of E protein residues involved in binding to AG129 mouse brain MRPs we utilized our panel of Asibi and 17D i.c.-derived chimeric viruses.

17D residues E-200, E-299, and E-305 were each able to induce binding to mouse brain when substituted into the Asibi backbone [E-200 (1.6 log_10_ reduction, *p* < 0.0001), E-299 (1.5 log_10_ reduction, *p* < 0.0001) and E-305 (3.3 log_10_ reduction, *p* < 0.0001)] but did not bind when the Asibi residues at those positions were exchanged in the 17D backbone [E-200 (0.5 log_10_ reduction, *p* = 0.0021) E-299 (0.7 log_10_ reduction, *p* < 0.0001), and E-305 (0.4 log_10_ reduction, *p* = 0.047)] (Table 1). Thus, residue E-305 appears critical to 17D virus binding to mouse brain MRPs.

The exchange of E-170, E-173, and E-407 did not affect the binding of Asibi to brain [E-170 (0.8 log_10_ reduction, *p* < 0.0001), E-173 (0.09 log_10_ reduction, *p* = 0.81), and E-407 (0.5 log_10_ reduction, *p* = 0.0083)] but did ablate the ability of 17D to bind brain MRP [E-170 (0.4 log_10_ reduction, *p* = 0.040), E-173 (0.2 log_10_ reduction, *p* = 0.35), and E-407 (0.2 log_10_ reduction, *p* = 0.44)]. The data for Asibi/17D E-170 were equivocal. Though the reduction in titer was statistically significant, the virus bound <1.0 log_10_, making it not significant by our definition.

The exchange of E-325 and E-380 did alter the ability of Asibi to bind brain MRP [E-325 (1.1 log_10_ reduction, *p* < 0.0001) and E-380 (2.0 log_10_ reduction, *p* < 0.0001)] and increased the binding of 17D to brain [E-325 2.2 log_10_ reduction, *p* < 0.0001) and E-380 (3.3 log_10_ reduction, *p* < 0.0001)].

E-52 did not affect binding of Asibi (0.4 log_10_ reduction, *p* = 0.013) nor 17D (1.1 log_10_ reduction, *p* < 0.0001) to brain MRP.

### Mapping residues involved in binding to mouse brain and liver on the E protein

**Fig. 4:**
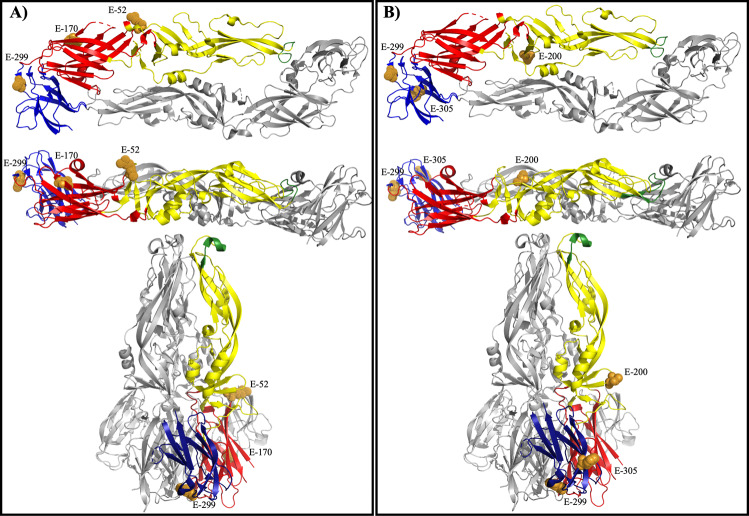
Mapping residues important to mouse liver MRP and mouse brain MRP binding to YFV E protein. Residues important to the YFV interact with AG129 liver MRP in both Asibi and 17D backbones were mapped to the prefusion E protein structure and displayed from the top and side view as well as the post-fusion structure (**A**). Residues important to the YFV interact with AG129 brain MRP in both Asibi and 17D backbones were mapped to the prefusion E protein structure and displayed from the top and side view as well as the post-fusion structure (**B**).

All three residues (E-52, E-170, E-299) important to YFV binding to mouse liver MRP clustered on the outside of the E protein dimer when viewed from the top, and extended past the virion surface when the E protein dimer is viewed from the side (Fig. 4A). Residue E-407 was also important to the binding of YFV to mouse liver MRP; however, E-407 is in the stem-anchor region of the E protein rather than in the ectodomain and therefore is not included in published crystal structures of the protein^38^. In particular, E-52 is the most exposed mature virus. In the post-fusion form of the YFV E protein, E-52 and E-170 are exposed whereas E-299 is not (Fig. 4A)

When the residues (E-200, E-299, and E-305) were mapped onto the prefusion YFV E protein dimer, it is clear that these residues are exposed on the surface of the virion (Fig. 4B). In the post-fusion form, E-200 and E-305 are outward-facing but E-299 is not exposed (Fig. 4B).

## Discussion

The YFV 17D vaccine has been successfully used to control YF disease for over 80 years. It was generated through serial passage of WT strain Asibi in mouse and chicken tissue, ending with passage in chick embryo lacking nervous tissue^2^. 17D virus differs from Asibi virus at 20 amino acid residues. Eight of the 20 residues reside in the E protein and are located in all three domains in the N-terminal ectodomain plus the transmembrane domain. In addition, E-325 is part of the 17D-204 vaccine substrain-specific epitope^18^.

The MRP technique assesses how well a “ligand” on the surface of the virus binds to cell membranes from a specific organ, but not how the virus causes disease once it enters the tissue. The technique for which the MRP protocol is modeled was used to identify binding sites of ligands to neurotransmitter receptors and biological function in terms of second signal transduction^19,39^. The incorporation of AG129 mouse lung as a control demonstrated the specificity of virus binding to MRPs from different tissues. We showed that Asibi virus bound to AG129 mouse liver but not brain MRPs, and 17D-204 virus the reverse, which is consistent with the AG129 mouse virulence phenotypes of WT Asibi and 17D-204 viruses^7^. We used prM/E and EDIII chimeras of 17D-204 and Asibi viruses to confirm that the E protein, and more specifically EDI/EDII mediate the changes in the ability of WT Asibi to bind mouse liver MRPs and EDIII mediates binding of 17D-204 vaccine to mouse brain MRPs. These results are consistent with previous studies that have shown that the E protein is a major determinant of flavivirus pathogenicity. EDI, responsible for linking EDII and EDIII, has been shown to influence neurovirulence of both JEV and DENV in mouse models^40–42^. EDII, the dimerization domain containing the fusion loop, includes two YFV type-specific epitopes (recognized by mAbs 2E10 and B39) and the only known WT YFV epitope, defined using the YFV mAb 117 used in this study^43–45^. The YFV type-specific epitopes included residues E-71/72/125 and E-153/155, respectively^43,44^. A previous study used plaque-purified mutants from 17D-204 to identify E-173 in this WT epitope^18^, one of the eight residues that distinguishes Asibi from 17D. EDIII, an Ig-like domain, has been implicated in receptor binding with regions of the upper lateral surface shown to be important to a 17D-204 substrain-specific epitope, mouse neurovirulence, and rate of viral clearance in mice^18,37,46^. The 17D-204 specific vaccine epitope was mapped to E-305 and E-325 within EDIII with mAb 864 and was shown to be distinct from the vaccine-specific mAb 411 used here through direct competition assays^18,47^. Although not biologically active, i.e., displaying no neutralizing activity, mAbs 117 and 411 can help to elucidate how structural changes affect regions of the E protein that influence attenuation and virulence.

**Fig. 5:**
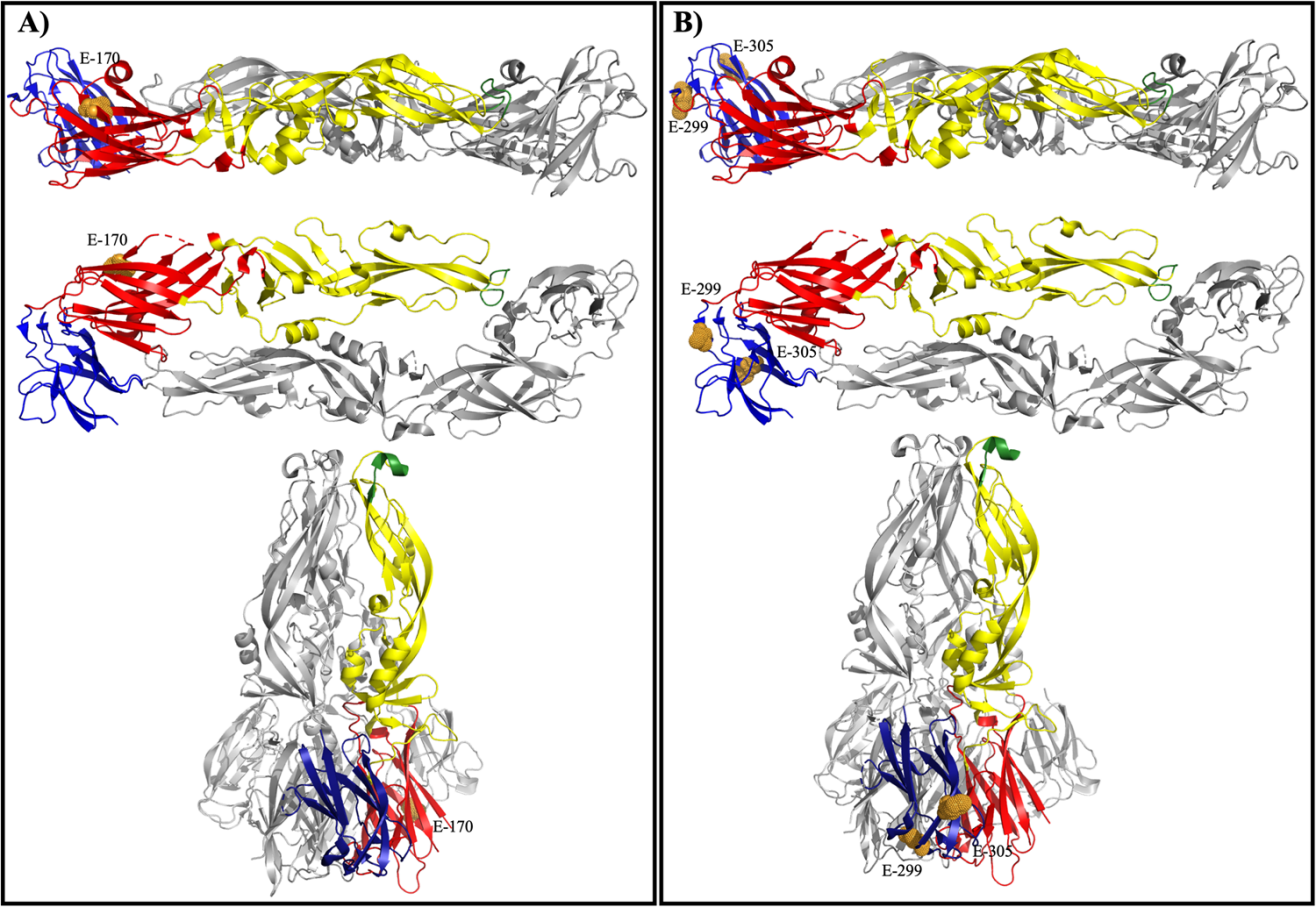
mAb epitopes and MRP binding domains overlap on YFV E protein. Residues important to the WT mAb 117/liver MRP binding (**A**) and vaccine mAb 411/brain MRP binding (**B**) were mapped onto the E protein prefusion (displayed from side and top) and post-fusion structures.

MAb 117 bound to residues in EDI and EDII, which confirms previous findings that E-173 was crucial to this epitope^45^. Using single-site mutants, we further defined the WT epitope to include residues M-36, E-170, E-173, and E-200 (see Fig. 3A and Table 1). In the 17D i.c. backbone, Asibi residues E-52, E-170, E-173, E-299, and E-407 also appeared to be important to the WT epitope. It is apparent that residues involved in the mAb 117 epitope are also important to how the virus interacts with mouse liver (E-52, E-170, and E-299, with E-173 equivocal and possibly a peripheral residue in binding) and substitution of the WT epitope resulted in loss of virulence in a mouse model^45^ (Fig. 5A).

Although it has been proposed that the E protein of YFV is responsible for the tissue tropism and changes in virulence phenotypes of WT strain Asibi and 17D, previous studies have shown the flavivirus M protein to impact mAb resistance and receptor binding making the role of M-36 unsurprising in the current studies^20,48–52^. In addition, mAb 864, which recognizes an epitope on EDIII, immunoprecipitated prM along with E from virus-infected cells suggesting its importance to the 17D-204 substrain-specific epitope recognized by mAb 864^11^. Taken together, it seems clear that, as with most flavivirus E protein epitopes (see review^42^), the WT epitope recognized by mAb 117 is not contiguous but rather conformational, recognizing the E protein at residues spanning multiple domains. Significantly, based on the structural model of the YFV E protein, these residues are all accessible at the surface of the virion^38^.

The vaccine-specific mAb, 411, was shown to bind an epitope that was contained in EDIII. The 17D-204 substrain epitope recognized by mAb 864 is also in EDIII but competition binding assays showed mAbs 411 and 864 do not recognize overlapping epitopes^18,53^. Specifically, mAb 411 binding involves 17D residues E-299, E-305, E-325, and E-380 (see Fig. 3B). As mAb 411 is vaccine-specific it also recognizes FNV, which does not display common residues at E-299, E-305, E-325, or E-380. In fact, at these residues, FNV displays common amino acids to the WT strain Asibi, which mAb 411 does not recognize. Taken together, this suggests that this epitope is conformational on the structure of the protein. In addition to its involvement in the vaccine-specific mAb epitope, EDIII was responsible for the binding of 17D to AG129 mouse brain tissue (specifically, E-299, E-305, and E-380), which is consistent with previous MRP studies^21,22^. In JEV mutants resistant to mouse brain MRPs, a residue change at E-306 (corresponding to E-305 in YFV) was responsible for neuroattenuation in mice^21,22^. The Asibi i.c. chimera with the 17D residue at E-305 bound to mouse brain MRP more tightly than 17D i.c. confirming the importance of this residue to the mouse brain MRP epitope. Asibi i.c. bound mouse brain MRP more strongly than 17D i.c. chimeras with Asibi residues at positions E-170 and E-173, which is interesting considering the 17D with the WT epitope recognized by mAb 117 could still cause neurotropic disease. In addition, a 17D-204 vaccine revertant isolated from a case of post-vaccinal encephalitis had mutations at E-155 (a glycosylation site) in EDII and E-303 in EDIII, which further supports a critical role of EDIII for neurovirulence. In support of our current findings, the neurovirulence phenotype of a neuroadaptated 17D strain was mapped to E-326 and E-380, two residues that were also shown to be integral to binding of virus to glycosaminoglycans, plus viral spread and virulence in mice^37,54,55^. Finally, we have previously shown that mutation of either E-305 or E-325 of 17D-204 vaccine attenuated mouse neurovirulence^18^.

Taken together, results here and from previous studies suggest that the vaccine-specific epitope recognized by mAb 411 (E-299, E-305, E-325, E-380) and 17D-204 specific epitope recognized by mAb 864 (E-305 and E-325) are physically located on EDIII and the same residues (E-299, E-305) are involved in 17D-204 vaccine virus binding to mouse brain MRPs (Fig. 5B). Structurally, the region where vaccine-specific epitope and mouse brain MRP binding residues overlap (E-299 and E-305) involves the EDIII loops that extend past the surface of the mature YFV virion. In the post-fusion structure of YFV E, E-305 is exposed whereas E-299 is buried in the bottom of the trimer spike. As these regions are generally accessible it is not surprising that multiple vaccine antibodies map to this region.

It is interesting that some of the residues important to WT and vaccine epitopes that also apparently control tissue tropism in AG129 mice overlap. Though not confirmed here, it is possible that these epitopes confer a change in virulence through the ability to bind pathogenically important organs. To date, no flavivirus receptor-binding molecule has been conclusively identified. However, 17D-204 has been shown to enter cells in a unique, clathrin-independent mechanism whereas WT Asibi virus utilizes the classical, clathrin-mediated endocytosis pathway^46^ supporting a role for differences in interactions of WT Asibi and 17D vaccine viruses with host cells. With the work presented here, it can be hypothesized that YFV interacts with different receptors on mouse liver and brain cells that involve EDI/II and EDIII, respectively. In the vaccine strain, this change could result in a severe adverse event (E-303), as seen with vaccine revertant P-16065^56^. It needs to be emphasized that this work was completed entirely in vitro and with mouse organs. Changes to receptors can have large impact on tissue tropism, virulence, and immune evasion, which may partially explain the attenuation of the 17D virus. Viscerotropism studies require non-human primates to obtain data directly relevant to humans, and requires strong ethical justification. The current studies are not at the stage yet. Nonetheless, we believe that the studies presented in mouse tissues could have relevance to other flavivirus infections, particularly neurotropic flaviviruses.

Finally, it is significant that these studies are not only applicable to YFV. Differing receptors for WT and vaccine strains has been shown for other viruses such as measles where WT strains CD46 to enter cells and vaccine strains utilize an unknown receptor, and reduced interaction with CD46^57,58^ suggesting that the phenotype of live-attenuated vaccines may be due in part to mutations in the surface glycoproteins that result in altered cell tropism. This area required further studies.

## Methods

### Viruses

Low passage Asibi virus was received from the late Dr. Robert Shope of the World Reference Center for Emerging Viruses and Arboviruses (Galveston, TX, Genbank: KF769016). The virus had been passaged six times in Rhesus macaques and three times in C6/36 *Aedes albopictus* mosquito cells to create a working stock. It has previously been shown to be lethal in NHPs^59^. The 17D-204 virus used in the studies was reconstituted, commercial dose of YFVAX™ (Sanofi-Pasteur, Lot#UF795AA) without passage.

I.c.s of both 17D-204 and Asibi viruses were used to generate all chimeric viruses, including single-site mutants used in these studies^25,26^. Hereafter, the 17D-204 infectious clone will be referred to as 17D i.c. Mutations to the genome were made using site-directed mutagenesis (QuikChange XL kit). Four micrograms of RNA was in vitro transcribed (Amplicap SP6 Message Maker) and transfected into Vero cells using standard procedures^27,60^. When virus-infected cells displayed 80% cytopathic effect, virus was harvested, and titrated in Vero cells using focus forming assay (FFA) as previously described^27^. RNA was extracted and genomes sequenced using Illumina methods to confirm the clone’s genetic identity and ensure no compensatory mutations had arisen (UTMB Sequencing Core). Single-site infectious clone mutants were generated at each common location that 17D substrains and Asibi differ in the E protein and in E-325 in both the 17D and Asibi backbones because this is a well-characterized 17D-204 substrain-specific neutralizing epitope^18^ (Table 1).

### Indirect immunofluorescence microscopy

Vero cells were inoculated at a multiplicity of infection (MOI) of 0.1 or mock-infected with PBS and incubated for 60 h, trypsinized, and seeded onto Teflon-coated spot slides (Polysciences) for 5 h at 37 °C. Cells were washed with PBS, fixed with 1:1 acetone:methanol, and slides stored at −20 °C until staining.

Spot slides were blocked for 1 h using 5% normal goat serum (NGS) and 3% bovine serum albumin (BSA). After washing thoroughly with TBS, slides were incubated with primary antibody (YFV mAb 117^47^ or YFV mAb 411^11^, respectively) diluted 1:1000 for 2 h at room temperature. Slides were washed with TBS and incubated with, goat anti-mouse IgG Dylight 488 (Invitrogen) in the dark for 1 h. After washing with TBS, slides were mounted using antifade mounting media with DAPI (Vector Laboratories), allowed to cure at room temperature for 15 min and then stored at 4 °C overnight. Slides were imaged with a Olympus BX61 fluorescence microscope using ×40 lenses.

### Generation membrane receptor preparations (MRPs)

MRPs were generated as previously described^24^. Briefly, brain, liver, and lung were collected from uninfected, 6–8-week-old, female AG129 mice. The organs were homogenized and the homogenate spun at 2000 × *g* for 10 min. The supernatant was collected and spun at 40,000 × *g* for 15 min. The resulting pellet was resuspended at 30 mg/mL in storage media (250 mM sucrose, 5 mM magnesium chloride, 50 mM Tris) and spun at 40,000 × *g* for 10 min. The resulting MRP was flash-frozen using liquid nitrogen. Aliquots were stored at −80 °C until use.

### Animal ethics statement

The animal study was conducted in accordance with the recommendations in the Guide for the Care and Use of Laboratory Animals of the National Institutes of Health. Animal protocol was approved by the Institutional Animal Care and Use Committee (IACUC) at the University of Texas Medical Branch (UTMB).

### MRP assay

MRP assays were undertaken in duplicate as previously described^20–24^. Briefly, MRP aliquots were rapidly thawed at 37 °C and kept on ice. Virus was mixed with MRP or control storage media at a 1:9 ratio. MRP and the control consisting of virus in storage media only were shaken using a Qiagen TissueLyser at 3 Hz for 1 h at room temperature. Samples were then centrifuged for 10 min at 12,000 × *g* in order to pellet any virus bound to MRP. The supernatant, containing residual, unbound virus, was titrated in Vero cell monolayers. Fold change and percentage binding were calculated by comparing the titer of MRP supernatant to the titer of virus in storage media only. MRP-resistant mutants were isolated for 17D and Asibi viruses through infecting Vero cell monolayer with supernatant from MRP assays (MRP-resistant [MRP^R^] portion) and plaques were picked from the resulting infection. Plaque picks were amplified once and titrated in Vero cells.

### Sanger sequencing of the E protein gene

RNA from aliquots of each plaque-picked virus was extracted and structural genes amplified using RT-PCR with previously published primers^61^. Sanger sequencing of purified PCR reactions was completed at the UTMB Molecular Genomics Core.

### Viral qRT-PCR

Viral RNA was extracted from stocks of the Asibi and 17D biological and i.c. viruses. cDNA was generated using iScript cDNA synthesis kit (Bio-Rad) and the qRT-PCR assay was completed using the iQ SYBR Green Supermix kit (Bio-Rad) and CFX96 real-time PCR system (Bio-Rad). Primers were directed at a region of the C protein where Asibi and 17D are identical (Forward: GCC GTT CCC ATG ATG TTC TG, Reverse: CAC CCG TCA TCA ACA GCA TT)^46^.

### Structural analysis of E protein

The location of E protein epitopes and residues important to MRP binding were displayed using pre- (PDB accession number: 6IW4) and post-fusion (PDB accession number: 6IW1) structures of the 17D E protein and Pymol software. The domains of one monomer were colored red (EDI), yellow (EDII), and blue (EDIII). The fusion loop of the same monomer was colored green.

### Reporting summary

Further information on research design is available in the Nature Research Reporting Summary linked to this article.

## Supplementary information

Supplementary Tables 1 and 2

Reporting Summary

## Data Availability

All unique biological materials and the corresponding datasets generated and analyzed during the current study are available from the corresponding author on reasonable request.
