# Supplementary material for: Impact of yellow fever virus envelope protein on wild-type and vaccine epitopes and tissue tropism
Source: NPJ Vaccines. 2022 Mar 23;7:39. doi: 10.1038/s41541-022-00460-6 (PMC8942996; doi:10.1038/s41541-022-00460-6)
Supplement: Supplementary file 1 — Supplementary Tables 1 and 2 [file 41541_2022_460_MOESM1_ESM.docx]

**Supplementary Table 1: Binding of Asibi and 17D biological and infectious clones to AG129 liver, brain and lung MRPs**

Panel A: mouse liver MRPs, Panel B: mouse brain MRPs and Panel C: mouse lung MRPs.

To facilitate interpretation of the results, binding data are presented as log_10_ reduction in infectivity in MRP samples when compared to controls, fold-change in infectivity titer, and percent loss in infectivity titer (i.e. percentage of infectious virus bound to MRPs). Significance is reported here is based on a Dunnett’s multiple comparisons test.

A)

| Liver MRP |  |  |  |  |  |
| --- | --- | --- | --- | --- | --- |
| **Virus** | **Log_10_change** | **Fold change** | **Percent binding** | **Significance** | **p-value** |
| Asibi i.c. | 1.8 | 59.3 | 98.3 | **** | <0.0001 |
| Asibi | 1.4 | 26.5 | 96.2 | **** | <0.0001 |
| 17D i.c. | 0.4 | 2.6 | 61.5 | ns | 0.25 |
| 17D-204 | 0.7 | 4.6 | 78.1 | ns | 0.22 |

B)

| Brain MRP |  |  |  |  |  |
| --- | --- | --- | --- | --- | --- |
| **Virus** | **Log_10_change** | **Fold change** | **Percent binding** | **Significance** | **p-value** |
| Asibi i.c. | 0.4 | 2.7 | 63.3 | ns | 0.98 |
| Asibi | 0.2 | 1.6 | 35.5 | ns | 0.99 |
| 17D i.c. | 1.9 | 84.4 | 98.8 | ** | 0.0023 |
| 17D-204 | 2.2 | 158.0 | 99.4 | *** | 0.0001 |

C)

| Lung MRP |  |  |  |  |  |
| --- | --- | --- | --- | --- | --- |
| **Virus** | **Log_10_change** | **Fold change** | **Percent binding** | **Significance** | **p-value** |
| Asibi i.c. | -0.6 | 0.2 | -3.3% | ns | 0.65 |
| Asibi | -0.05 | 0.9 | -11.9 | ns | 0.99 |
| 17D i.c. | 0.6 | 0.2 | 3.3% | ns | 0.46 |
| 17D-204 | -0.2 | 0.7 | -33.0 | ns | 0.22 |

**Supplementary Table 2: Sequencing of MRP^R^ structural genes**

Plaque picks were performed on the supernatant of MRP assays to generate MRP^R^ viruses. The structural genes of the resulting viruses were sequenced and compared to the input virus. Three brain MRP^R^ viruses were generated and two liver MRP^R^ viruses were generated for both Asibi i.c. and 17D i.c. Some amino acid changes were not detected (n.d.) in that particular MRP^R^

**A)**

|  |  |  | AG129 brain MRP^R^ | | AG129 liver MRP^R^ | |
| --- | --- | --- | --- | --- | --- | --- |
| **Gene** | **Codon within Gene** | **Amino Acid-**  **Asibi i.c** | **Amino Acid- MRP^R^** | **Plaque containing mutation (out of 3)** | **Amino Acid- MRP^R^** | **Plaque containing mutation (out of 2)** |
| E | 56 | A | n.d. | n.d. | V | p.p. 1, p.p. 2 |
| E | 170 | A | n.d. | n.d. | V | p.p. 1, p.p. 2 |
| E | 173 | T | n.d. | n.d. | I | p.p. 1, p.p. 2 |
| E | 200 | K | n.d. | n.d. | T | p.p. 1, p.p. 2 |
| E | 394 | S | T | p.p. 1 | n.d. | n.d. |

**B)**

|  |  |  | **AG129 brain MRP^R^** | | **AG129 liver MRP^R^** | |
| --- | --- | --- | --- | --- | --- | --- |
| **Gene** | **Codon within**  **Gene** | **Amino Acid –**  **17D i.c.** | **Amino Acid-**  **MRP^R^** | **Plaque containing mutation (out of 3)** | **Amino Acid-**  **MRP^R^** | **Plaque containing mutation (out of 2)** |
| E | 56 | V | n.d | n.d. | A | p.p. 1, p.p. 2 |
