# Supplementary material for: Impact of yellow fever virus envelope protein on wild-type and vaccine epitopes and tissue tropism
Source: NPJ Vaccines. 2022 Mar 23;7:39. doi: 10.1038/s41541-022-00460-6 (PMC8942996; doi:10.1038/s41541-022-00460-6)
Supplement: Supplementary file 2 — Reporting Summary [file 41541_2022_460_MOESM2_ESM.pdf]

nature research

Corresponding author(s): Barrett,Alan

Last updated by author(s): Jul 9, 2021

Reporting Summary

Nature Research wishes to improve the reproducibility of the work that we publish. This form provides structure for consistency and transparency in reporting. For further information on Nature Research policies, see our Editorial Policies and the Editorial Policy Checklist.

Statistics

For all statistical analyses, confirm that the following items are present in the figure legend, table legend, main text, or Methods section.

n/a	Confirmed	
☐	☒	The exact sample size (n) for each experimental group/condition, given as a discrete number and unit of measurement
☐	☒	A statement on whether measurements were taken from distinct samples or whether the same sample was measured repeatedly
☐	☒	The statistical test(s) used AND whether they are one- or two-sided<br>*Only common tests should be described solely by name; describe more complex techniques in the Methods section.*
☒	☐	A description of all covariates tested
☐	☒	A description of any assumptions or corrections, such as tests of normality and adjustment for multiple comparisons
☒	☐	A full description of the statistical parameters including central tendency (e.g. means) or other basic estimates (e.g. regression coefficient) AND variation (e.g. standard deviation) or associated estimates of uncertainty (e.g. confidence intervals)
☐	☒	For null hypothesis testing, the test statistic (e.g. F, t, r) with confidence intervals, effect sizes, degrees of freedom and P value noted<br>*Give P values as exact values whenever suitable.*
☒	☐	For Bayesian analysis, information on the choice of priors and Markov chain Monte Carlo settings
☒	☐	For hierarchical and complex designs, identification of the appropriate level for tests and full reporting of outcomes
☒	☐	Estimates of effect sizes (e.g. Cohen's d, Pearson's r), indicating how they were calculated

Our web collection on statistics for biologists contains articles on many of the points above.

Software and code

Policy information about availability of computer code

Data collection	Not applicable
Data analysis	not applicable

For manuscripts utilizing custom algorithms or software that are central to the research but not yet described in published literature, software must be made available to editors and reviewers. We strongly encourage code deposition in a community repository (e.g. GitHub). See the Nature Research guidelines for submitting code & software for further information.

Data

Policy information about availability of data

All manuscripts must include a data availability statement. This statement should provide the following information, where applicable:

- Accession codes, unique identifiers, or web links for publicly available datasets
- A list of figures that have associated raw data
- A description of any restrictions on data availability

There is no raw data in any of the figures except for Figure 2 that shows indirect immunofluoresence. The data that support the findings of this study are available from the corresponding author upon reasonable request.

Field-specific reporting

Please select the one below that is the best fit for your research. If you are not sure, read the appropriate sections before making your selection.

☒ Life sciences ☐ Behavioural & social sciences ☐ Ecological, evolutionary & environmental sciences

For a reference copy of the document with all sections, see nature.com/documents/nr-reporting-summary-flat.pdf

Life sciences study design

All studies must disclose on these points even when the disclosure is negative.

Sample size	not applicable
Data exclusions	not applicable
Replication	Replicates: MRP binding experiments were completed twice. The binding data presented is the mean of these two replicates. Multiple plaque picks (three for liver MRP resistant viral plaque picks and two for brain MRP resistant viral plaque picks) were completed and sequenced to confirm the sequences detected were integral to MRP resistance.
Randomization	not applicable
Blinding	not applicable

Reporting for specific materials, systems and methods

We require information from authors about some types of materials, experimental systems and methods used in many studies. Here, indicate whether each material, system or method listed is relevant to your study. If you are not sure if a list item applies to your research, read the appropriate section before selecting a response.

Materials & experimental systems

n/a	Involved in the study	
☐	☒	Antibodies
☐	☒	Eukaryotic cell lines
☒	☐	Palaeontology and archaeology
☐	☒	Animals and other organisms
☒	☐	Human research participants
☒	☐	Clinical data
☒	☐	Dual use research of concern

Methods

n/a	Involved in the study	
☒	☐	ChIP-seq
☒	☐	Flow cytometry
☒	☐	MRI-based neuroimaging

Antibodies

Antibodies used	Monoclonal antibody (mAb) 117 is a YFV WT-specific antibody. MAb 411 is YF vaccine specific antibody
Validation	Validation was completed in the following references:<br>Barrett, A. D. et al. Identification of monoclonal antibodies that distinguish between 17D-204 and other strains of yellow fever virus. J Gen Virol 71 (Pt 1), 13 18 (1990).<br>Gould, E. A. et al. Examination of the immunological relationships between flaviviruses using yellow fever virus monoclonal antibodies. J Gen Virol 66 (Pt 7), 1369 1382 (1985).<br>Ledger, T. N. et al. Variation in the biological function of envelope protein epitopes of yellow fever vaccine viruses detected with monoclonal antibodies. Biologicals 20, 117 128 (1992).<br>Gould, E. A., Buckley, A., Cane, P. A., Higgs, S. & Cammack, N. Use of a monoclonal antibody specific for wild-type yellow fever virus to identify a wild-type antigenic variant in 17D vaccine pools. J Gen Virol 70 (Pt 7), 1889 1894 (1989).<br>Cammack, N. & Gould, E. A. Topographical analysis of epitope relationships on the envelope glycoprotein of yellow fever 17D vaccine and the wild type asibi parent virus. Virology 150, 333–341 (1986).

Eukaryotic cell lines

Policy information about cell lines

Cell line source(s)	Vero cells from ATCC
Authentication	Authentication completed by ATCC

Mycoplasma contamination	All viruses grown in the Vero cells were subjected to Next Generation Sequencing using random heximer primers. No mycobacterial contamination was identified
Commonly misidentified lines (See ICLAC register)	*Name any commonly misidentified cell lines used in the study and provide a rationale for their use.*

Animals and other organisms

Policy information about studies involving animals; ARRIVE guidelines recommended for reporting animal research

Laboratory animals	brain, liver and lung were obtained from three deceased uninfected AG129 mice
Wild animals	Not applicable
Field-collected samples	Not applicable.
Ethics oversight	UTMB IACUC

Note that full information on the approval of the study protocol must also be provided in the manuscript.
